# Supplementary material for: Ethephon-Induced Ethylene Enhances Protein Degradation in Source Leaves, but Its High Endogenous Level Inhibits the Development of Regenerative Organs in Brassica napus
Source: Plants (Basel). 2021 Sep 23;10(10):1993. doi: 10.3390/plants10101993 (PMC8537263; doi:10.3390/plants10101993)
Supplement: Supplementary file 1 [file plants-10-01993-s001.zip › plants-1374272-supplementary.pdf]

SUPPLIMENTARY TABLE S1. Primer sequences used for qRT-PCR analysis.

**Gene**	**Accession No.**	**Forward sequence (5'-3')**	**Reverse sequence (5'-3')**
CAB	AY288914	GGCAGCCCATGGTACGGATC	CCTCCITCGCTGAAGATCTGT
SAG12	XM_013821610.2	AGAGAATACCAAACCAAACCGAA	GCAACTCCCAAAATCTCAGGG
AAP1	AY188953.1	TGCTTACGCCACGGTTCTCA	GCTGCGCGAACACCTGATAG
AAP2	AY188954.1	CGGTACTGTTTGGACCGCAA	GACACGGGTCTTTTCCTCCG
AAP4	AY188955.1	CGCTGGACCTGCAGTGATGT	CCGCTCTCGTGAAAGCAGTTT
AAP6	AJ565848.1	GCGGCCGTAATGTCCTTTTC	ACCCACACACCCGCATAACA
ACTIN	AF111812	GATTCCGTTGCCCTGAAGTA	GCGACCACCTTGATCTTCAT
